# Puerperal Fever

**Published:** 1873-05-01

**Authors:** 


### MEDICAL EXAMINER.

*A Semi-Monthly Journal of Medical Sciences.*

EDITED BY

N. S. DAVIS, M. D., AND F. H. DAVIS, M. D.

Chicago, May 1st, 1873.

### EDITORIAL.

### PUERPERAL FEVER.

One of our subscribers asks us to give the results of our clinical observations in the treatment of puerperal fever, in the columns of THE EXAMINER. We cannot possibly command the time to do this satisfactorily at the present time. Inasmuch as the disease is quite prevalent in some parts of the State, we will hastily express some thoughts that may not be wholly without practical value. Among the gleanings from our exchanges, in the present number, will be found the conclusions deduced by some of the most recent investigators in regard to the special pathology of this much dreaded disease.

They represent it as always caused by septicæmic poison, generally absorbed through some abrasion or injury, either in the vaginal mucous membrane or in the uterus. It is supposed that such poison may be developed by putrefactive changes in the retained blood coagula and vaginal secretions; and its absorption induces a specific inflammation of the uterine vessels and peritoneum. This view of the origin and pathology of puerperal fever may be correct in a large proportion of cases; but we are sure that in many seasons there is something more than the mere local action of a septic poison. There is an epidemic influence, or general condition of the atmosphere, which, if it is not capable of causing the disease, independent of the absorption of local poison, must create a very unusual tendency to rapid putrefactive or septic changes in the retained blood and secretions in the vagina and uterus. Certain it is that a practitioner often passes a number of years without meeting a single case of puerperal fever, and then encounters a season when a large proportion of all who are confined within the circle of his acquaintance suffer from it. It is not reasonable to suppose that the women confined in such seasons are more injured during their labor, or that any more blood or coagula are retained than those confined in years when no case of the disease is seen. So far as our observation goes, the seasons of special prevalence of puerperal fever are the same in which we also have either erysipelas, cerebro-spinal meningitis, or typhus. And this leads to a suggestion of great practical importance. It is that, in seasons and neighborhoods when and where either of the diseases just named are specially prevalent, all pregnant women who are within three or four weeks of the time for their confinement, should not only take special pains to promote a healthy condition of the blood by proper out-door air, plain diet, special cleanliness of person, and good ventilation of sleeping rooms, but they should take, daily, either the tincture of chloride of iron or sulphite of soda, in such doses as would establish a moderate influence of these agents in the blood. And these same remedies should be continued, still more actively, during the first week or ten days after the the confinement. We are confident that these simple precautions would prevent attacks of the disease in a very large proportion, if not all, of the cases. Another prophylactic measure should be alluded to. It is very common for women and nurses to keep the lying-in room closely curtained and dark, especially during the first week. This we deem always wrong, and especially so in a season of epidemic tendency. Light is essential to health, and more or less *sun-light* should be admitted into every lying-in chamber as certainly as the sun shines. In regard to the treatment of puerperal fever, our observation teaches us that no absolute rules or routine can be adopted for our guide. We have had cases in which we have bled freely within the first six hours after the initial rigor, and followed the bleeding by full doses of opium combined with alterative doses of calomel, alternated with veratrum viride for the first two or three days, and the patients have recovered.

We have had other cases in which bleeding has been omitted, and the patient kept fully under the influence of opium and the sulphites for the first two days; and, when the abdomen became tympanitic, full doses of oil of turpentine have been added, sometimes quinia, and success has attended this method. We think that this latter method, judiciously carried out, with proper attention to vaginal cleanliness and abdominal fomentations, is the best that can be pursued in a great majority of cases.

Venesection, to have any chance of benefit, must be practiced very *early* and decisively. Free application of leeches to the hypogastrium may be practiced with more impunity in the first stage, then general bleeding.

